# Supplementary material for: Implications of the three-dimensional chromatin organization for genome evolution in a fungal plant pathogen
Source: Nat Commun. 2024 Feb 24;15:1701. doi: 10.1038/s41467-024-45884-x (PMC10894299; doi:10.1038/s41467-024-45884-x)
Supplement: Supplementary file 3 — Reporting Summary [file 41467_2024_45884_MOESM3_ESM.pdf]

nature portfolio

Corresponding author(s): prof. dr. Bart Thomma

Last updated by author(s): 20-01-2024

Reporting Summary

Nature Portfolio wishes to improve the reproducibility of the work that we publish. This form provides structure for consistency and transparency in reporting. For further information on Nature Portfolio policies, see our Editorial Policies and the Editorial Policy Checklist.

Statistics

For all statistical analyses, confirm that the following items are present in the figure legend, table legend, main text, or Methods section.

n/a	Confirmed	
☐	☒	The exact sample size (*n*) for each experimental group/condition, given as a discrete number and unit of measurement
☐	☒	A statement on whether measurements were taken from distinct samples or whether the same sample was measured repeatedly
☐	☒	The statistical test(s) used AND whether they are one- or two-sided<br>*Only common tests should be described solely by name; describe more complex techniques in the Methods section.*
☐	☒	A description of all covariates tested
☐	☒	A description of any assumptions or corrections, such as tests of normality and adjustment for multiple comparisons
☐	☒	A full description of the statistical parameters including central tendency (e.g. means) or other basic estimates (e.g. regression coefficient) AND variation (e.g. standard deviation) or associated estimates of uncertainty (e.g. confidence intervals)
☐	☒	For null hypothesis testing, the test statistic (e.g. *F*, *t*, *r*) with confidence intervals, effect sizes, degrees of freedom and *P* value noted<br>*Give P values as exact values whenever suitable.*
☒	☐	For Bayesian analysis, information on the choice of priors and Markov chain Monte Carlo settings
☒	☐	For hierarchical and complex designs, identification of the appropriate level for tests and full reporting of outcomes
☐	☒	Estimates of effect sizes (e.g. Cohen's *d*, Pearson's *r*), indicating how they were calculated

Our web collection on statistics for biologists contains articles on many of the points above.

Software and code

Policy information about availability of computer code

Data collection	No software was used for data collection
Data analysis	Trim sequencing reads: Trimmomatic v.0.36<br>Read mapping: BWA-MEM v.0.7.17. picard 2.23.9.<br>Hi-C data handling: HiCExplorer tools v.3.6, fanc v.0.9.20, HiCRep v.1.12.2.<br>ChIP-seq normalization: deepTools v.3.1.3, Peak calling homer v.4.11.<br>Genomic variation: GATK v.4.0, TEPID v.2.0, svcallers v.1.0, MUMmer v.3.0<br>Genomic conservation: Realphy v.1.12, ProgressiveCactus v.1.2.3, Phast v.1.5, Blastn 2.5.0.<br>Data processing in R.4.0.5. Umap-learn v.0.2.8.0. regionR v.1.18.1. Matplotlib, Numbpy, Searborn v.0.8.1. bedtools v.2.27.1.<br>Data visualization EnrichedHeatmap v.1.20, circlize v.0.4.15, ggplot2 v.3.4.1.

For manuscripts utilizing custom algorithms or software that are central to the research but not yet described in published literature, software must be made available to editors and reviewers. We strongly encourage code deposition in a community repository (e.g. GitHub). See the Nature Portfolio guidelines for submitting code & software for further information.

Data

Policy information about availability of data

All manuscripts must include a data availability statement. This statement should provide the following information, where applicable:

- Accession codes, unique identifiers, or web links for publicly available datasets
- A description of any restrictions on data availability
- For clinical datasets or third party data, please ensure that the statement adheres to our policy

The Hi-C, ChIP-seq, RNA-seq, ATAC-seq and Bisulfite-seq data used in this study are available in the NCBI database under the Bioproject accession codes PRJNA592220 and PRJNA641329 [https://www.ncbi.nlm.nih.gov/bioproject/?term=PRJNA592220, https://www.ncbi.nlm.nih.gov/bioproject/?term=PRJNA641329]. The genome sequence of the refer-ence Verticillium dahliae isolate JR2 and VdLs17 used in this study is available at NCBI under the accession codes GCA_000400815.2 and GCA_000952015.1, respectively [https://www.ncbi.nlm.nih.gov/datasets/genome/GCA_000400815.2, https://www.ncbi.nlm.nih.gov/search/all/?term=GCA_000952015.1]. Source Data is available at https://doi.org/10.5281/zenodo.10579548.

Research involving human participants, their data, or biological material

Policy information about studies with human participants or human data. See also policy information about sex, gender (identity/presentation), and sexual orientation and race, ethnicity and racism.

Reporting on sex and gender	N/A
Reporting on race, ethnicity, or other socially relevant groupings	N/A
Population characteristics	N/A
Recruitment	N/A
Ethics oversight	N/A

Note that full information on the approval of the study protocol must also be provided in the manuscript.

Field-specific reporting

Please select the one below that is the best fit for your research. If you are not sure, read the appropriate sections before making your selection.

[x] Life sciences [ ] Behavioural & social sciences [ ] Ecological, evolutionary & environmental sciences

For a reference copy of the document with all sections, see nature.com/documents/nr-reporting-summary-flat.pdf

Life sciences study design

All studies must disclose on these points even when the disclosure is negative.

Sample size	No sample size calculation was performed as each sample represented a strain of a single species.
Data exclusions	No data was excluded from the study.
Replication	Two biological replicates of each strain, or single species, were grown on potato-dextrose broth media. Liquid cultures were grown for four days in the dark at 22ºC and 160 RPM. Each biological replicate of the fungal cultures was used for further Hi-C and ChIP-seq analyses. The normalized average of the two biological replicates was used.
Randomization	No phenotypical assessments were performed in the experimental design. Each sample represented a strain of a single species. Thus, randomization is not relevant for this study.
Blinding	No phenotypical assessments were performed in the experimental design. Thus, blinding is not relevant for this study.

Reporting for specific materials, systems and methods

We require information from authors about some types of materials, experimental systems and methods used in many studies. Here, indicate whether each material, system or method listed is relevant to your study. If you are not sure if a list item applies to your research, read the appropriate section before selecting a response.

Materials & experimental systems

n/a	Involved in the study
☒	☐ Antibodies
☒	☐ Eukaryotic cell lines
☒	☐ Palaeontology and archaeology
☒	☐ Animals and other organisms
☒	☐ Clinical data
☒	☐ Dual use research of concern
☒	☐ Plants

Methods

n/a	Involved in the study
☐	☒ ChIP-seq
☒	☐ Flow cytometry
☒	☐ MRI-based neuroimaging

Plants

Seed stocks: N/A

Novel plant genotypes: N/A

Authentication: N/A

ChIP-seq

Data deposition

☒ Confirm that both raw and final processed data have been deposited in a public database such as GEO.

☒ Confirm that you have deposited or provided access to graph files (e.g. BED files) for the called peaks.

Data access links
May remain private before publication.

https://www.ncbi.nlm.nih.gov/bioproject/PRJNA592220
https://www.ncbi.nlm.nih.gov/bioproject/PRJNA641329

Files in database submission

PRJNA59220: SRX7253439, SRX7253438, SRX7253437, SRX7253436, SRX7253435, SRX7253434, SRX7253433, SRX7253432, SRX7253431, SRX7253430, SRX7253429, SRX7253428, SRX7253427, SRX7253426, SRX7253425, SRX7253424, SRX7253423; PRJNA641329: SRX8602312, SRX8602311, SRX8602310, SRX8602309, SRX8602308, SRX8602307, SRX8602306, SRX8602305, SRX8602304, SRX8602303, SRX8602302, SRX8602301, SRX8602300, SRX8602299, SRX8602298, SRX8602297, SRX8602296, SRX8602295, SRX8602294, SRX8602293, SRX8602292, SRX8602291

Genome browser session
(e.g. UCSC)

Not applicable

Methodology

Replicates

We used already publicly available data. Two biological replicates on potato-dextrose broth media were performed. Liquid cultures were frown for four days in the dark at 22ºC and 160 RPM. The normalized average of two biological replicates was used.

Sequencing depth

Single-end (125 bp) sequence reads. Genome size 36142631. Uniquely mapped reads: 1667367 (H3K4me2); 9495874 (H3K27ac); 8669997 (H3K27me3); 3080302 (H3K9me3).

Antibodies

Catalog number #39913 #39765 #39155, #39134 ActiveMotif; Carlsbad, California USA.

Peak calling parameters

We used already publicly available data. Reads were mapped to the V. dahliae JR2 reference genome using BWA-mem with default settings. Three regions of the genome were masked due to aberrant mapping (Ch1:1-45000, Chr2:3466000-3475999, Chr3:1-4200). Mapped reads were RPGC-normalized using deepTools bamCoverage –binSize 10 –smoothLength 30. Then, input-control signal was subtracted from normalized values, and negative values were set to 0. Peak calling was performed using HOMER findpeaks --style histone with default settings

Data quality

We used already publicly available data. Correlation between replicates was verified by principal components analysis and by Pearson's correlation. For each consensus sample we obtained 6898 (H3K4me2), 3593(H3K27ac), 15194(H3K27me3), 3121 (H3K9me3) variable size peaks, with a p-value of 0.0004 and 2x fold enrichment.

Software

Mapping using BWA-MEM v.0.7.17. Normalization deepTools v.3.1.3. Peak calling homer v.4.11. Further data processing in R.4.0.5.
